# Noninvasive imaging diagnosis of sinusoidal obstruction syndrome: a pictorial review

**DOI:** 10.1186/s13244-019-0791-x

**Published:** 2019-11-20

**Authors:** Yun Zhang, Yuling Yan, Bin Song

**Affiliations:** 1 Department of Radiology, Sichuan University West China Hospital, No.37, Guoxue Alley, Chengdu, 610041 Sichuan Province China; 2 Department of Gastroenterology and Hepatology, Sichuan University West China Hospital, Chengdu, 610041 Sichuan Province China

**Keywords:** Sinusoidal obstruction syndrome, Sinusoidal endothelial cells, Diagnostic criteria, Imaging studies, Budd-Chiari syndrome

## Abstract

Sinusoidal obstruction syndrome (SOS) is a rare liver disorder due to hepatic vascular injury. Its rapid and accurate diagnosis is crucial for patient survival. SOS is often established clinically, based on Baltimore, modified Seattle, or European Society for Blood and Marrow Transplantation (EBMT) criteria. Unfortunately, such criteria are not highly specificity and fail to provide a timely, reliable differential diagnosis. The use of noninvasive imaging techniques, such as ultrasound (US), computed tomography (CT), magnetic resonance imaging (MRI), and fluorodeoxyglucose positron emission tomography/computed tomography (FDG-PET/CT), has recently grown in this setting, some key imaging features offering diagnostic improvement. This review provides a synopsis of current noninvasive imaging techniques used for this purpose, summarizing accurate and reliable diagnostic features of SOS.

## Key points

- Rapid and accurate diagnosis is crucial for SOS patient’s survival.
- Hepatic hemodynamic changes and parenchymal heterogeneity are the characteristic features of SOS.
- Cellular dysfunction and portal hypertension-related complications contribute to the differential diagnosis of SOS.
- Noninvasive imaging diagnosis offering diagnostic improvement of SOS.

## Introduction

Sinusoidal obstruction syndrome (SOS), also referred to as veno-occlusive disease, is a rare and highly lethal condition of the liver due to vascular injury. Hematopoietic stem cell transplantation (HSCT) is most often implicated, but SOS is also associated with drug toxicity. Common agents include medicinal herbs containing pyrrolidine alkaloids (PAs), antineoplastic drugs (e.g., 6-thioguanine, methotrexate, and 6-mercaptopurine), and cytotoxic chemotherapies for treating liver metastases [1–5].

SOS is typically diagnosed on a clinical basis, using Baltimore [6], modified Seattle [7, 8], or European Society for Blood and Marrow Transplantation (EBMT) criteria [9, 10]. Hepatomegaly, ascites, and bilirubin elevation are the chief diagnostic parameters, although these clinical criteria are not highly specific [5]. In addition, severe SOS may rapidly progress to multiorgan failure before a clinical diagnosis is apparent, prohibiting timely intervention and therapeutic control. Thus, more rapid and accurate diagnostic criteria are urgently needed.

Liver biopsy is the gold standard method for confirmation of SOS. However, this approach is invasive and carries a high risk of infection, rendering its routine use impractical. The recent use of noninvasive imaging techniques, including ultrasound (US), computed tomography (CT), magnetic resonance imaging (MRI), and fluorodeoxyglucose positron emission tomography-computed tomography (FDG-PET/CT), has grown in this setting given the improved diagnostics of key imaging features [11–13]. This review is focused on pertinent imaging advancements, summarizing distinctive elements that enable an accurate and reliable diagnosis of SOS.

### Pathology and pathophysiology of SOS

**Fig. 1:**
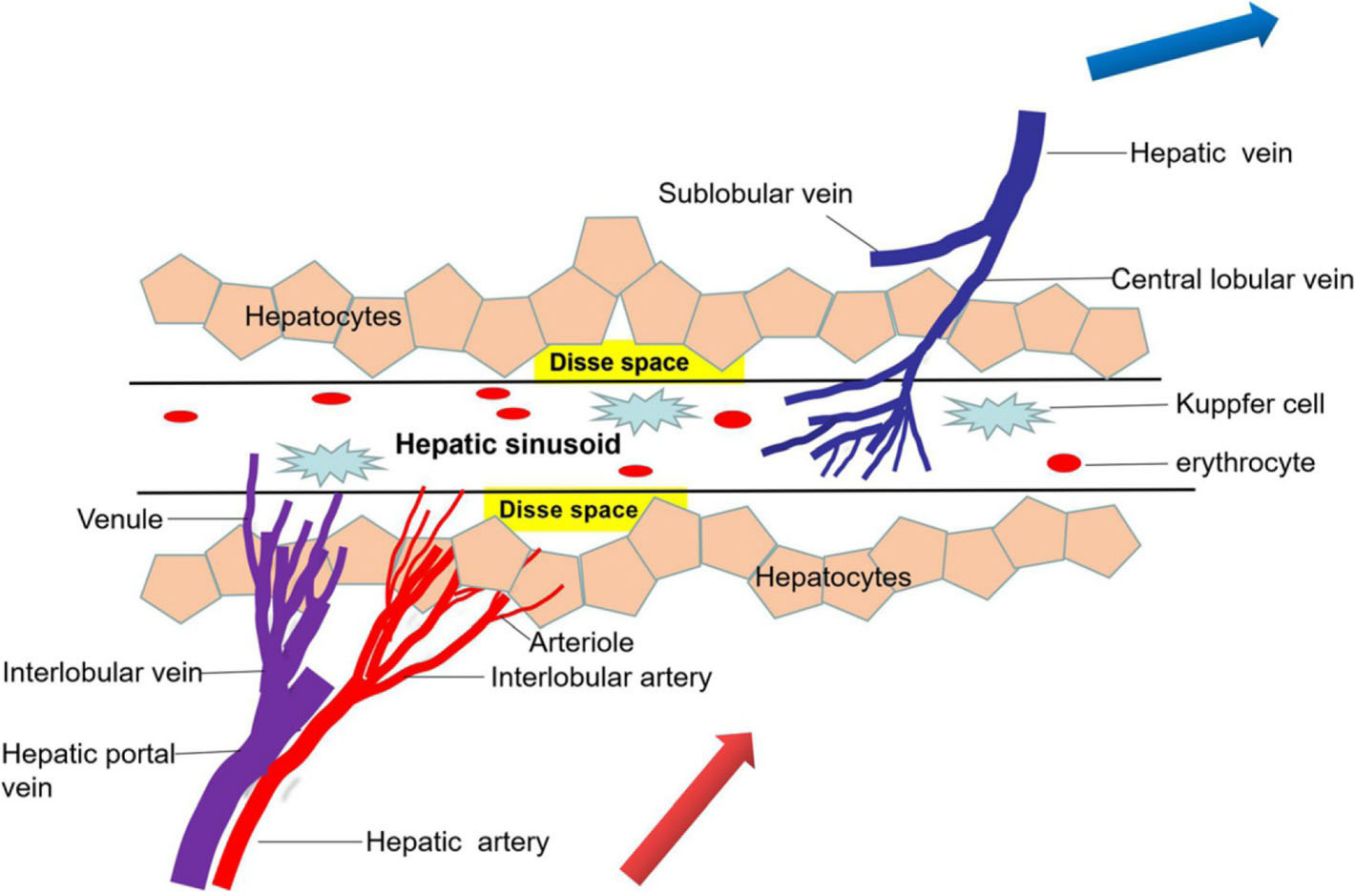
Hepatic sinusoid blood flow diagram. Hepatic sinusoids are a complex of vascular conduits to be responsible for the exchange of blood, oxygen, and nutrients in the liver and systemic circulation. The structure of the hepatic sinusoids is composed of the sinusoidal endothelial cells and containing Kupffer cell. Under normal circumstances, the blood supply to the hepatic sinusoid blood come from the inlet flows (i.e., the hepatic artery and portal vein) and venous blood return to the heart through the outlet flows (i.e., the hepatic veins)

SOS was first reported in 1979 as an early and serious complication of HSCT [14]. It was attributed to sinusoidal endothelial cell cytotoxicity induced by serial conditioning treatments prior to HSCT [15]. Drug toxicity damage includes chemotherapy drugs for patients with liver metastases of colorectal cancer and PA-imbued medicinal herbs (such as Tu san qi) have been increasingly reported in recent decades. A central pathogenic event of SOS is toxic damage of a hepatic sinusoidal endothelial cell, marked primarily by cleavage of sinusoidal endothelial cells and substantial congestive sinusoidal dilatation in centrilobular areas and perisinusoidal fibrosis. Sinusoidal endothelial cell injury ultimately eventuates in endothelial cell exfoliation of the terminal hepatic vein, erythrocytic decomposition, and a glut of hemosiderin within the space of Disse, promoting sinusoidal blood flow impeded and leading to portal hypertension syndrome and hepatocytes dysfunction (Fig. 1).

### Limitations of clinical diagnostic criteria for SOS

Baltimore [6] and modified Seattle criteria [8] are generally used to establish a clinical diagnosis of SOS. The chief clinical parameters are hyperbilirubinemia, hepatomegaly, jaundice, ascites, or unexplained weight gain. The European Society for Blood and Marrow Transplantation (EBMT) published separate criteria for adults and children, and the update is the first to acknowledge US-determined hemodynamic change as a reliable metric of SOS. Moreover, the Chinese Society of Gastroenterology Committee of Hepatobiliary Disease has addressed a void in terms of managing PA-related SOS by issuing the Nanjing criteria (2019) [16]. However, some instances of SOS are late in onset, and there is low disease specificity, an array of disorders presenting similarly. Thus, regardless of the improvements made and the broader applicability of established criteria, the diagnostic specificity of these criteria (rooted in clinical symptoms) is still problematic, so false-positive rates are unavoidably high.

### Noninvasive imaging findings of SOS

Imaging modalities, including US, CT, MRI, and FDG-PET/CT, have become the first-line methods in the diagnosis of liver disease. Some exploratory studies conducted in recent years have focused on the diagnostic potential of imaging technologies in this setting. Key imaging features of SOS have been identified that provide more timely and convincing evidence of SOS, compared with clinical parameters. Furthermore, there are state-of-the-art functional MRI technologies and quantitative imaging methods capable of predicting functional damage at a cellular level, based on intrinsic hepatic changes (Table 1).

**Table 1:** Different imaging modalities for SOS and their findings

Major imaging modalities in diagnosis of SOS			
Modality		Role	Imaging features of SOS
US	B-mode US	Screening and early differential diagnosis, especially in asymptomatic or late-onset cases	Hepatosplenomegaly, gallbladder wall thickening > 6 mm, portal diameter > 12 mm, hepatic vein diameter < 3 mm, and indirect signs suggest portal hypertension such as ascites and visualization of collateral circulatory
	Doppler US	Surveillance and diagnostic, revealing morphologic changes, and flow velocity fluctuation	Hepatic vein diameter < 3 mm, collateral circulatory visualization, demodulation of portal vein flow, spectral density decline, congestion index < 0.1, portal vein flow < 10 cm/s, hepatic artery resistive index > 0.75, and monophasic flow in hepatic veins
	CEUS	Rapid diagnosis and differential diagnosis	Diffuse or geographic enhancement of hepatic parenchyma, with a scattering of hypoecho areas; hypoechoic lesion with hypervascularity in the arterial phase and a rapid wash-out appearance in the portal phase
CT		Diagnostic	Cloverleaf or claw-like shapes; lesion with peritumoral enhancement and central low attenuation
MRI	All gadolinium-enhanced MRI	Diagnostic	Cloverleaf or claw-like shapes; peritumoral enhancement lesion with central low-signal intensity
	Gadoxetate-enhanced MRI	Diagnostic and provide information about liver function	Diffuse or geographic hypo-intensity
	SWI	Differential diagnosis	Geographic or nodular hypo-intensity

*SOS* sinusoidal obstruction syndrome, *US* ultrasound, *CEUS* contrast-enhanced ultrasound, *CT* computed tomography, *MRI* magnetic resonance imaging, *SWI* susceptibility-weighted imaging

#### Hepatic hemodynamic changes

As an important means of monitoring blood flow, US offers more information for early and differential diagnosis of SOS, especially in asymptomatic or late-onset cases [17]. Some findings of B-mode US, including hepatosplenomegaly, gallbladder wall thickening > 6 mm, portal diameter > 12 mm, hepatic vein diameter < 3 mm, and indirect signs suggest portal hypertension such as ascites and visualization of collateral circulatory, have been used as a routine criteria for SOS diagnosis. Doppler technique is a noninvasive method to improve the diagnostic efficiency of B-mode US by revealing morphologic changes and flow velocity fluctuation in hepatic vessels [18]. At present, the known characteristics of Doppler US of SOS include hepatic vein diameter < 3 mm, collateral circulatory visualization, demodulation of portal vein flow, spectral density decline, congestion index < 0.1, portal vein flow < 10 cm/s, hepatic artery resistive index > 0.75, and monophasic flow in hepatic veins. These hemodynamic changes are closely associated with the course of disease progression. At the early stage of the disease, the sinusoidal outflow tract has a smooth blood flow allowing portal vein to maintain a normal blood flow and morphology; however, as the disease progress, hepatic venous blood flow disappears and stenosis of the main hepatic veins and portal vein blood flow is reduce and reflux occurs in some severe cases [6, 19, 20]. Dynamic monitoring of changes in the above may help predict disease progression and venture a prognosis of patients with SOS. Also, three-dimensional structures of hepatic blood vessels may be viewed using multidetector CT (MDCT)/multi-planar reformation (MPR) in combination and liver CT angiography (CTA). A disordered intrahepatic vascular network, an enlarged hepatic artery, ill-defined small arteries, and invisible hepatic veins are regular features of SOS [21, 22]. Recently, some researchers have applied FDG-PET/CT to further ascertain functional changes of the liver due to altered hepatic hemodynamics. Compared with baseline images, a rise (~ 10%) in standard uptake value of the liver (SUV_liver_) was observed after the onset of SOS. Presumably, sinus endothelial cell injury produced altered hemodynamics and microcirculatory obstruction, disrupting inlet/outlet flow patterns in hepatic vessels. Such changes then induce hepatic congestion and passively increase hepatic blood-pool FDG tracer activity [23].

#### Hepatic parenchymal heterogeneity

**Fig. 2:**
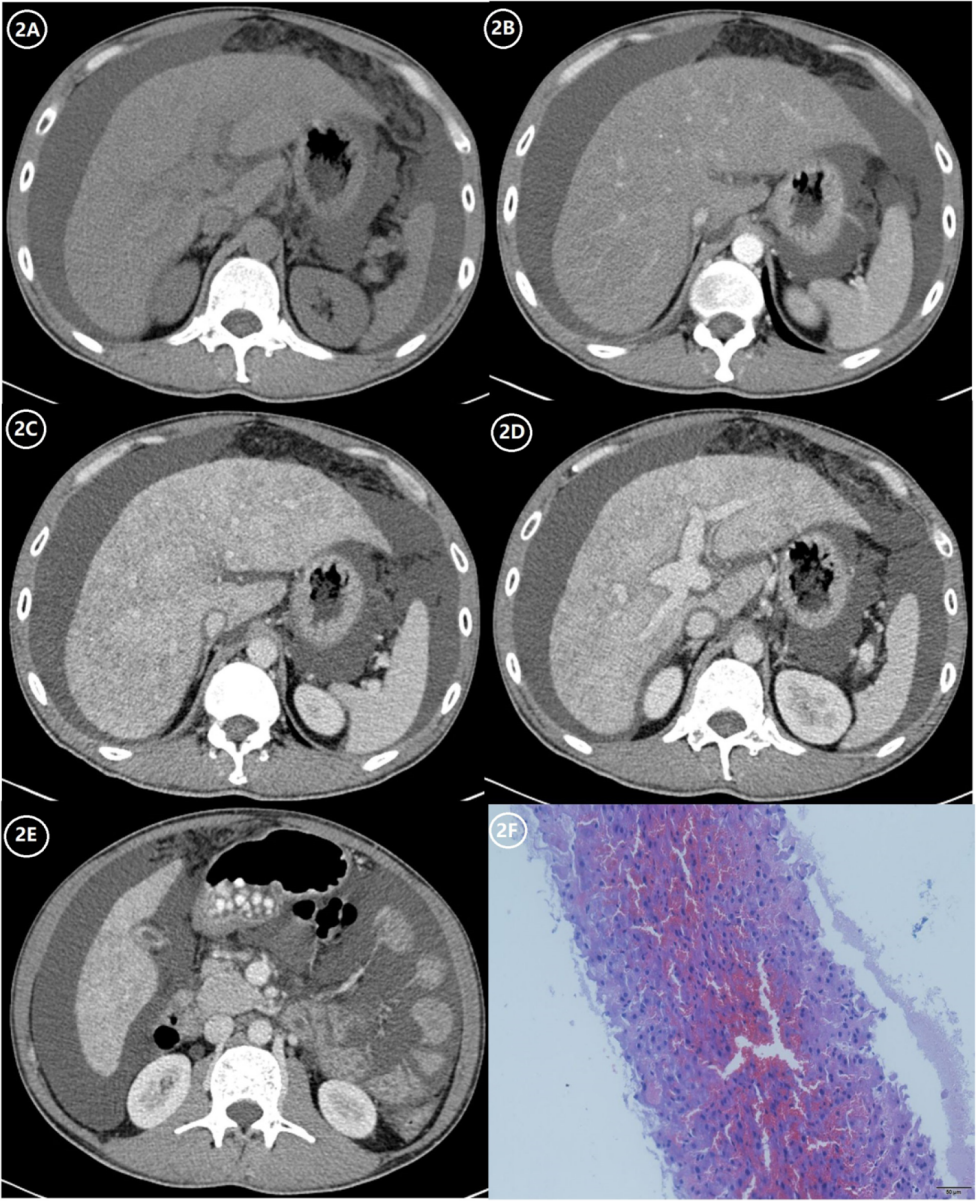
A 39-year-old man with SOS caused by Tu san qi. **a** Normal CT scan shows non-uniform density of the liver parenchyma, hepatomegaly, and medium to a large amount of ascites. **b** Arterial phase contrast-enhanced CT scan shows non-uniform enhancement of hepatic parenchyma. **c**–**e** Portal phase contrast-enhanced CT scan shows patchy low-density lesions with a non-uniform decrease of hepatic parenchyma enhancement (the CT value of the liver is lower than that of the spleen), portal hypertension combined with collateral circulation development, gallbladder wall edema, and edema around the portal vein. **f** The hematoxylin-eosin (HE) staining at × 200 magnification shows expansion and congestion around the central hepatic vein and hepatic sinus, hepatocyte edema, and mild central venous fibrosis, which is confirmed to be SOS

**Fig. 3:**
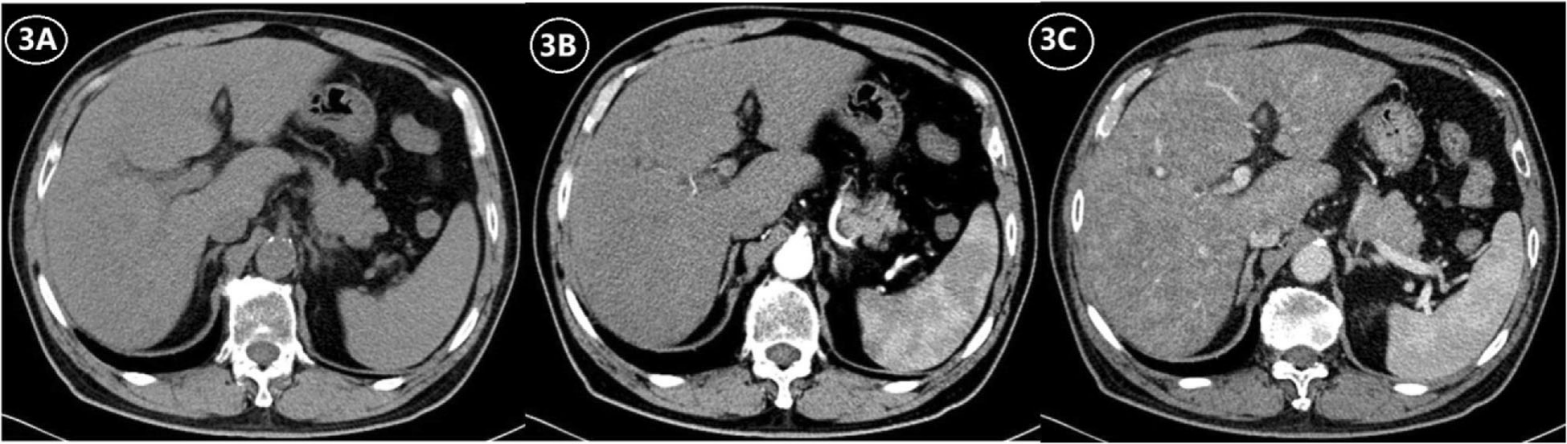
A 68-year-old man with SOS caused by Tu san qi. **a** Normal CT scan shows a non-uniform density of the liver parenchyma and mild hepatosplenomegaly. **b** Arterial phase contrast-enhanced CT scan shows a small and ill-defined hepatic artery and decrease of hepatic parenchyma enhancement. **c** Portal phase contrast-enhanced CT scan shows patchy low-density lesions with a non-uniform decrease of hepatic parenchyma enhancement (the CT value of the liver is lower than that of the spleen)

**Fig. 4:**
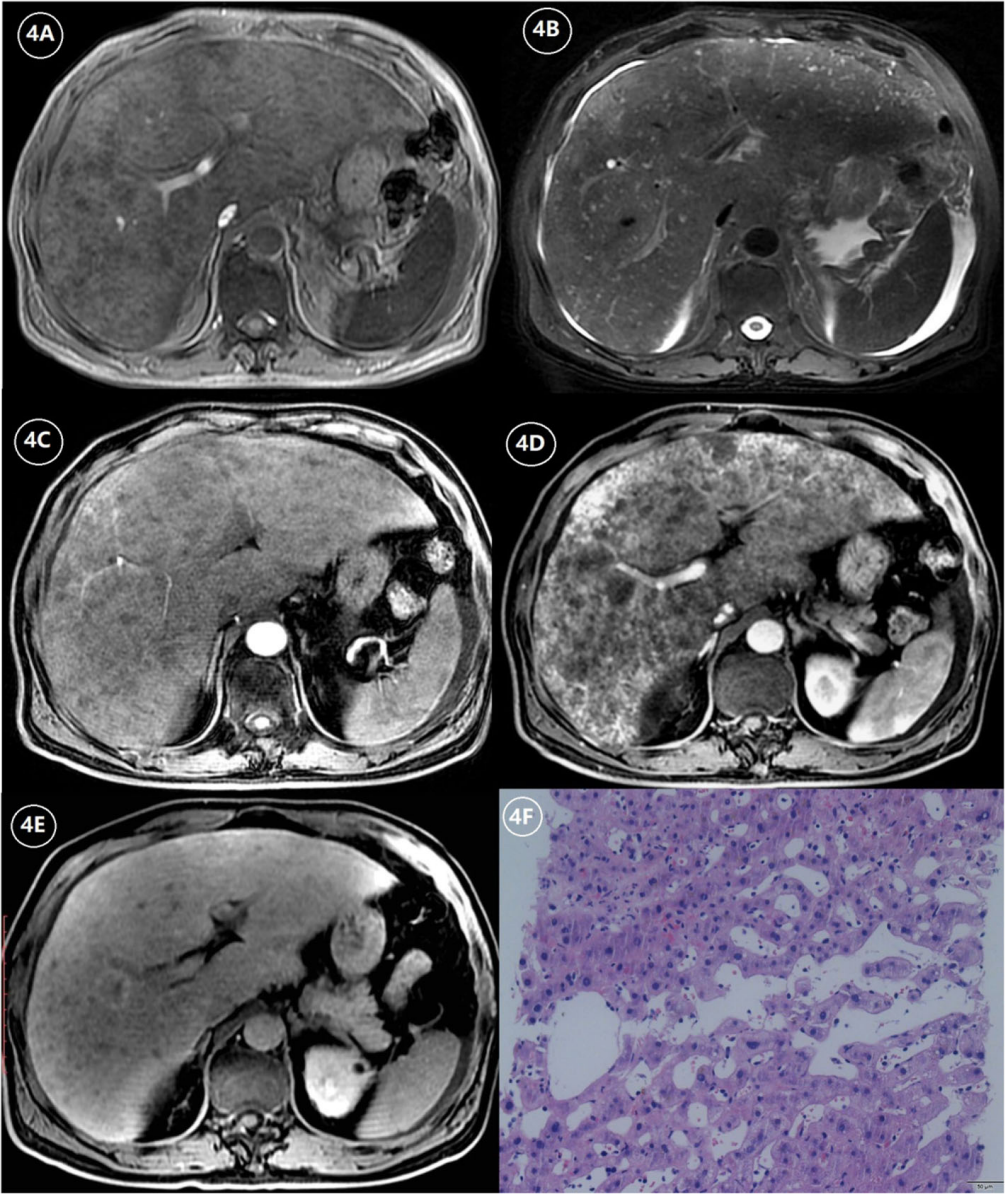
**a** The T1-weighted image (T1WI) shows hepatomegaly, a non-uniform intensity of liver parenchyma with a patchy and nodular low-signal intensity of the liver. **b** The T2-weighted image (T2WI) shows a non-uniform signal intensity of liver parenchyma with a patchy and nodular high-signal intensity of the liver and ascites. **c** Arterial phase contrast-enhanced MRI scan shows heterogeneous enhancement of liver parenchyma. **d** Portal phase contrast-enhanced MRI scan shows a significant heterogeneous enhancement of liver parenchyma with patchy and nodular lesions with decreased enhancement. **e** HBP of gadoxetic acid-enhanced MRI shows the signal intensity of liver parenchyma is slightly decreased and accompanied by the nodular lesions with lower-signal intensity. **f** The hematoxylin-eosin (HE) staining at × 200 magnification shows hepatocyte edema and significant hepatic sinus expansion, which is confirmed to be SOS

**Fig. 5:**
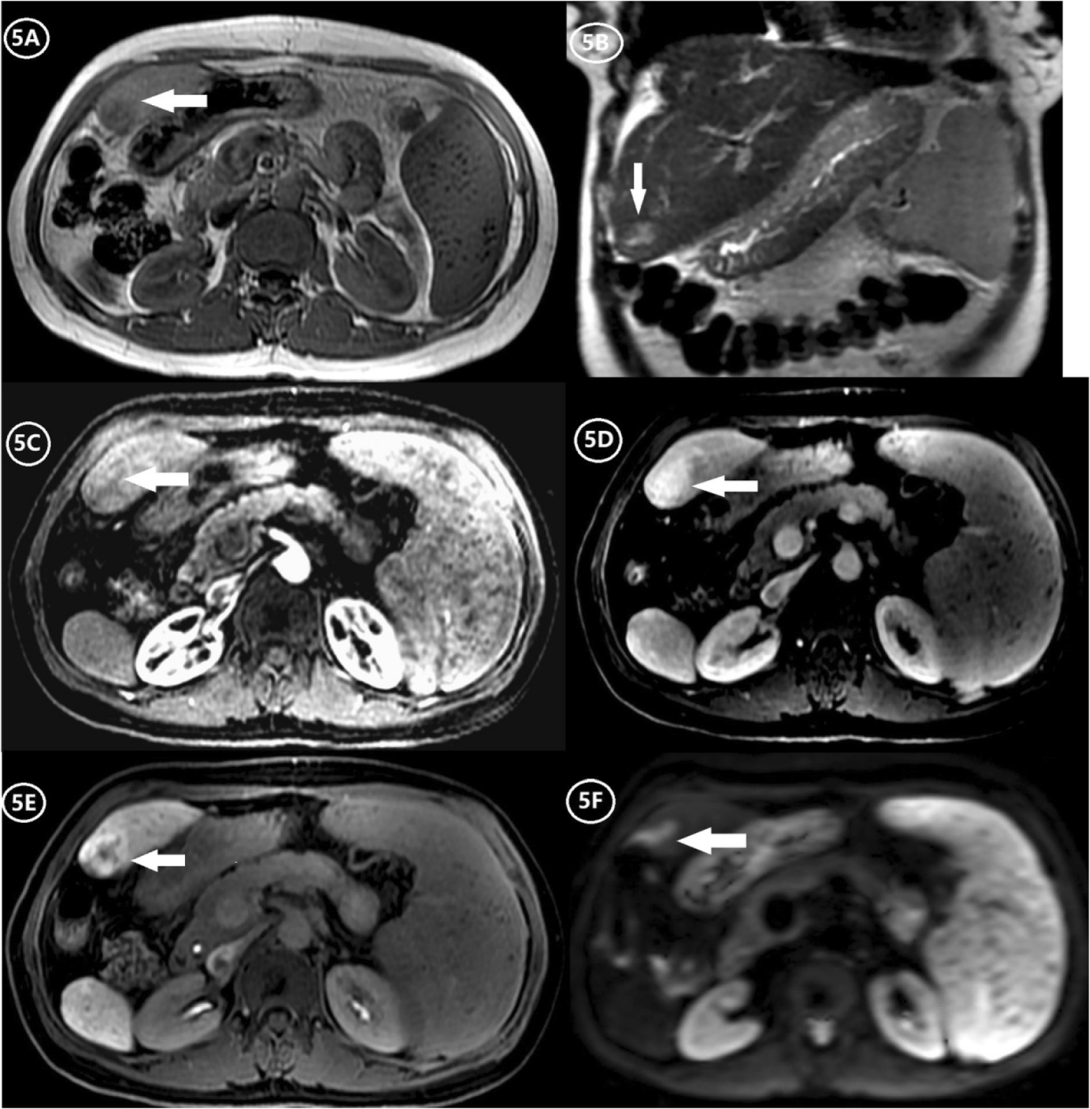
A 48-year-old woman with focal SOS (arrows) developed by chemotherapy after hepatectomy. **a**, **b** A nodular lesion in liver segment III with low-signal intensity on the T1-weighted image (T1WI) and high-signal intensity on the T2-weighted image (T2WI). **c** Arterial phase contrast-enhanced MRI scan shows a nodular lesion in liver segment III with mild peripheral enhancement. **d** Portal phase contrast-enhanced MRI scan shows the nodular lesion with apparent, uniform, and persistent enhancement. **e** HBP of gadoxetic acid-enhanced MRI shows the nodular lesion with a low-signal intensity at the center and high-signal intensity around it, which presents as “focal nodular hyperplasia” like lesion. **f** The diffusion-weighted imaging (DWI) shows an apparent limited diffusion

Diffuse, patchy, or geographic heterogeneity of hepatic parenchyma, with a scattering of hypodense areas, is typical of SOS (Figs. 2, 3, and 4). Past studies indicate that parenchymal heterogeneity scores positively correlate with SOS clinical severity [13, 24, 25]. In patients with hepatic metastases of colon cancer, the efficacy of oxaliplatin-based chemotherapy is impacted by the existing SOS, and the severity of parenchymal heterogeneity is closely associated with a poor tumor response [26, 27]. Among all the heterogeneity features on enhanced CT or MRI images, cloverleaf or claw-like shapes are two classic manifestations of SOS [28]. These manifestations refer to the markedly enhanced area around the main hepatic veins which shows increased blood supply compared to the normal liver parenchyma. The appearance of characteristic shapes perhaps related to the small vascular channels surrounding the main hepatic veins. Because the initial lesion of SOS is located in hepatic sinusoid, which makes central lobular vein and sublobular vein become the first to be involved. As the disease worsens, obvious edema and necrosis of adjacent hepatocytes lead to the decrease of hepatic parenchymal enhancement; however, the small vascular channels adjacent to the main hepatic vein are kept unobstructed and result in the increase of enhancement of the liver parenchyma adjacent to the venules [12]. Recently, the hepatic pseudotumor of SOS has been noted after oxaliplatin treatment [22, 29]. Lesion of this sort is pathologically characterized by sinusoidal dilatation and congestion of hepatocytes with inflammatory cellular infiltration and areas of fibrosis [30]. On liver imaging, pseudotumor of SOS may simulate focal nodular hyperplasia, atypical HCC, or liver metastases because of the variety of enhancement patterns due to the pathological changes in the disease progression [30] (Fig. 5). On contrast-enhanced US, pseudotumor of SOS usually presents as an ill-defined hypoechoic lesion with a uniform and obvious hypervascularity in the arterial phase and a rapid wash-out appearance in the portal phase. On multi-phase dynamic-enhanced CT or MRI, an irregular lesion with peritumoral enhancement and central low attenuation/signal intensity is the most common manifestation of pseudotumor of SOS. For further improving differential diagnostic efficiency between pseudotumor of SOS and malignancy, a qualitative imaging modality, such as diffuse-weighted imaging (DWI) is recommended, which plays a role in the theoretical basis of the cellular density difference [23].

#### Impairment of cellular function

Susceptibility-weighted imaging (SWI) is a quantitative imaging method that exploits differences in magnetic sensitivity to evaluate tissue densities. At present, SWI is advantageous in assessing various pathologies, particularly hemorrhages, micro-bleeds, or iron deposition in the brain [31]. The pivotal event (i.e., endothelial cell injury) in SOS ultimately eventuates in erythrocytic decomposition and a glut of hemosiderin within the space of Disse, potentially detectable by SWI. Some researchers have tested this theory by adding SWI sequences to routine MRI studies. The patchy low-signal intensities of resultant SWI images seem to approximate a portal venous-phase distribution, thus confirming these pathologic underpinnings and affording a new and noninvasive means for early diagnosis of SOS [32]. MRI studies are frequently enhanced by superparamagnetic iron-oxide nanoparticles (SPION-MRIs) or gadolinium ethoxybenzyl diethylenetriamine pentaacetic acid (Gd-EOB-DTPA) to assess the function of hepatocytes. SPION-MRIs provide a quantitative gauge of damage to Kupffer cells in the course of SOS, exhibiting fair agreement between severity of SOS and its corresponding histopathology [33]. The hepatobiliary phase (HBP) of a Gd-EOB-DTPA-enhanced MRI is a better index of actual hepatocytic function. Previous investigations have shown that Gd-EOB-DTPA-enhanced MRIs deliver high specificity and good interobserver consistency in evaluating the progression of SOS [34]. Furthermore, Yoneda et al. have reported a relation between organic anion transporting polypeptides (OATP) and functionality of hepatocytes [13]. They have documented SOS-related functional damage, showing that low-signal intensities on HBP images correlate positively with degrees of hepatocytic injury. Of note, gradual hepatic functional recovery after interruption of chemotherapy is verifiable by continuous HBP scanning.

#### Liver fibrosis

Fibroscan testing and acoustic radiation force impulse (ARFI) imaging, the most popular noninvasive transient elastography technologies, have been widely used to assess ongoing hepatic fibrosis and cirrhosis. Liver stiffness measurements (LSMs) determined by Fibroscan correspond with changes in the mechanical properties of the liver. Clinical increases in LSMs are seen in many conditions, including inflammation, fibrosis, congestion, cholestasis, and portal hypertension [35–37]. The injury to sinusoidal endothelial cells inherent in SOS promotes endothelial cell embolization and sinusoidal obstruction, leading to subendothelial fibrosis of sinusoids and venules, and eventual congestive fibrosis of the liver. Thus, LSM elevations may be useful preclinical and surveillance biomarkers of SOS [38]. Indeed, earlier data suggest a pre-transplant LSMs cut-point of 8 kPa in predicting liver toxicity after HSCT, thus supporting the implementation of quantitative SOS diagnostics [39]. In addition, an animal experiment has indicated that liver shear-wave velocity (SWV), measured by ARFI imaging, bears a close relation to histologic scores of SOS, as well as degrees of hepatic lobular inflammation. Observed SWI outcomes were significantly higher in rats with SOS, as opposed to a matched control group [40]. Finally, hepatic SWV has been used in some studies (with promising results) as a quantitative biomarker to evaluate the treatment responses of patients with SOS [41].

#### Signs associated with portal hypertension

Hepatosplenomegaly, ascites, a thickened gallbladder wall, and periportal edema are the chief imaging features of SOS [42] (Fig. 2). Such manifestations are hallmarks of portal hypertension, stemming from obstructed intrahepatic venous drainage, and the events that follow (i.e., congestion of the liver and spleen, loss of liver compliance, and hepatic injury). Because many diseases are similar in terms of clinical and laboratory parameters, Erturk et al. consider narrowed hepatic veins and collateral aspects of portal hypertension to be the features distinguishing SOS from other conditions [43].

### Differential diagnosis of SOS

Differential diagnosis of SOS includes graft-versus-host-disease (GVHD) and other causes of hepatic venous outflow obstruction such as Budd-Chiari Syndrome (BCS). GVHD is the major complication of Allogeneic-HCST and is the leading cause of early mortality after transplantation [44]. The nature of GVHD is a T cell-mediated immune response. Although some clinical manifestations of GVHD are similar to that of SOS such as toxic reactions, weight gain, and deranged liver function tests; however, the distinctive features of GVHD are more often associated with inflammatory symptoms (e.g., pyrexia) and a series of skin illness (e.g., poikiloderma, nail dystrophy, and alopecia) [45]. At present, imaging studies have limitations in the diagnosis of GVHD, although some intestinal CT abnormalities have been found in acute GVHD after HCST in children [46]. BSC is an obstruction from hepatic veins to the superior end of the inferior vena cava (IVC). It is characterized by centrilobular congestion and parenchymal destruction but without the endothelial cell exfoliation of the terminal hepatic vein. The primary causes of BSC include hepatic vein or IVC thrombosis, intraluminal invasion by parasite or malignancy, and extraluminal compression by solid tumor or cyst [47]. Imaging studies play an indispensable role in providing a reliable differential diagnosis between BSC and SOS. Stenosis and obstruction of hepatic veins or IVC and the presence of abnormal blood flow signals of the intrahepatic veins observed in Doppler US show good diagnostic capabilities for BSC, with a specificity of nearly 85% [48]. On contrast-enhanced CT images, a “mottled appearance” along with late enhancement of the perihepatic regions of the liver and around the hepatic veins may help to differentiate SOS from BCS. This type of parenchymal heterogeneity may result from the presence of venous shunts that drain central areas and the caudate lobe of the liver. This is also the reason why an enlarged caudate lobe frequently observed in patients with BCS. MRI improves the diagnostic performance of BSC by showing collateral and a spider-web network pattern of IVC. Moreover, early parenchymal necrosis caused by BSC and abnormal perfusion of affected liver segments could be better displayed by MRI multiple sequence scans.

## Conclusion

SOS is a life-threating liver disorder related to vascular injury. At present, a timely and reliable diagnosis is unachievable using established, clinically based criteria. Recently, noninvasive imaging technologies have assumed growing importance in identifying occurrences of SOS, monitoring its progression, or predicting patient prognosis. Hemodynamic changes of hepatic vessels, hepatic parenchymal heterogeneity, impaired cellular function, liver fibrosis, and an array of findings signaling portal hypertension (i.e., hepatosplenomegaly, ascites, thickened gallbladder wall, and periportal edema) are all characteristic of SOS in noninvasive imaging studies. Coupled with clinical data, these imaging attributes of SOS may improve the diagnostic accuracy, especially in late-onset and asymptomatic cases, ensuring timely medical intervention.

Further prospective studies are needed to better gauge the diagnostic utility of imaging markers in terms of accuracy and specificity for SOS. Moreover, a comprehensive diagnostic and predictive model must be developed, incorporating clinical, etiologic, and imaging parameters.

## Data Availability

The datasets used during the current study are available from the corresponding author on a reasonable request.

## Abbreviations

ARFI: Acoustic radiation force impulse
BCS: Budd-Chiari syndrome
BCSH/BSBMT: British Committee for Standards in Hematology/British Society for Blood and Marrow Transplantation
CT: Computed tomography
CTA: CT angiography
DWI: Diffuse-weighted imaging
EBMT: European Society for Blood and Marrow Transplantation
FDG-PET/CT: Fluorodeoxyglucose positron emission tomography-computed tomography
Gd-EOB-DTPA: Gadolinium-ethoxy benzyl-diethylenetriamine pentaacetic acid
GVHD: Graft-versus-host-disease
HBP: Hepatobiliary phase
HSCT: Hematopoietic cell transplantation
IVC: Inferior vena cava
LSMs: Liver stiffness measurements
MENA: Middle East and North Africa
MPR: Multi-planner reformation
MRI: Magnetic resonance imaging
OATP: Organic anion transporting polypeptides
PAs: Pyrrolidine alkaloids
SOS: Sinusoidal obstruction syndrome
SPIONs: Superparamagnetic iron-oxide nanoparticles
SWI: Susceptibility-weighted imaging
SWV: Shear-wave velocity
US: Ultrasound
